# Using Ecological Momentary Assessment to Document and Investigate Caregiver Practices Between Pediatric Therapy Sessions: Prospective Pilot Cohort Study

**DOI:** 10.2196/83548

**Published:** 2026-07-03

**Authors:** Jennifer K James, Mary P Shotwell, Hannah M Jensen, Shirley A James

**Affiliations:** 1 Wichita State University, 1845 Fairmount Street, Wichita, KS, United States, 1 316-978-5776; 2 Rocky Mountain University of Health Professions, 1800 S Novell Place, Provo, UT, United States

**Keywords:** dosage, pediatric therapy, adherence, engagement, ecological momentary assessment, EMA

## Abstract

**Background:**

Determining the appropriate dosage of pediatric occupational therapy, physical therapy, and speech-language pathology services is important when supporting families of children with disabilities. However, therapy dosage is inconsistently reported, and caregiver-delivered practice between sessions is rarely documented. Ecological momentary assessment (EMA) offers a method to capture caregiver practice in real time and to examine factors that influence it.

**Objective:**

This study aims to pilot the use of EMA to measure caregiver practices between therapy sessions and to compare EMA-reported practices with caregiver recall.

**Methods:**

This pilot prospective cohort study used convenience sampling to recruit caregivers of children receiving therapy services. During September 2024, participants completed a confidential baseline Qualtrics survey in their homes, which included recall of home practice from the previous week. Participants were then invited to complete 30 days of EMA logging of daily practice. Five participants enrolled in the EMA phase, which began 24 to 72 hours after baseline survey completion and took place during October and November 2024. Semistructured follow-up interviews were conducted immediately after the 30-day EMA period.

**Results:**

Of the 34 survey participants, 5 continued to the EMA phase, contributing 150 days of data, with 82 completed entries (82/150, 55%). Caregivers primarily completed EMA logs on days when practice occurred; missing entries were coded as zero practice based on caregiver reports. Recalled practice averaged 4.5 (SD 5.65) bouts/day and 11.6 (SD 6.35) minutes/bout, totaling 71.2 (SD 121.02) minutes/day. EMA-reported practice across all days (n=150) averaged 2.7 (SD 4.39) bouts/day and 6.5 (SD 6.45) minutes/bout, totaling 23.2 (SD 14.12) minutes/day, which was substantially lower than recalled estimates. On days when practice was reported (n=82), EMA-documented practice averaged 5.2 (SD 3.28) bouts/day and 6.5 (SD 6.45) minutes/bout, totaling 23.9 (SD 14.72) minutes/day. Variability in recalled practice was high (mean 71.19, SD 121.02 min/d). Caregivers described practice as occurring in short, frequent bouts embedded within daily routines, with routine integration, child engagement, and recall of therapist strategies identified as key facilitators.

**Conclusions:**

Caregiver-delivered practice occurred in short, frequent bouts integrated into daily routines. EMA-reported practice was substantially lower than caregiver recall, suggesting that retrospective recall and prospectively reported EMA data may differ substantially. These findings highlight the importance of teaching strategies that are brief, engaging, and easily incorporated into daily routines. Despite the small sample, EMA was acceptable to a subset of caregivers who completed participation; however, substantial attrition between survey enrollment and EMA initiation suggests significant feasibility and participation barriers that warrant further investigation.

## Introduction

The appropriate dosage of pediatric occupational therapy, physical therapy, and speech-language pathology services (therapy) is an important area of research in pediatric rehabilitation [1-5]. Concerns about staffing shortages, changes in service provision following the COVID-19 pandemic, and emerging research demonstrating the importance of increased intensity of therapy services have led to concerns that many children, especially young children and children with complex diagnoses, may not receive adequate services to maximize their developmental progress [4,6-8]. Few studies address the practice that children receive between formal therapy sessions from their caregivers [1,2].

Understanding the impact of the caregiver’s actions on the dosage of child practice is an important component of determining the level of support children need to meet developmental outcomes [1,2]. One study estimated that as many as 93% of learning opportunities occur outside formal therapy sessions [9]. Many pediatric providers use caregiver coaching and other interaction strategies to promote increased the caregiver’s confidence and competence in using skilled intervention strategies between therapy sessions [1,8]. If coaching is successful, we should expect caregivers to provide significant amounts of practice for children between therapy sessions [1,8]. Caregiver-delivered practice is critical in determining the amount of support children and families need from pediatric therapists, and is important for determining the appropriate frequency, intensity, and duration of services children and families require.

Existing research suggests that caregivers’ use of strategies between therapy sessions (practice) typically occurs in response to joint planning with the provider to implement therapeutic strategies during daily routines and activities. The relevance of intervention strategies to family routines, caregiver engagement, child diagnosis, and family demographics likely influences caregiver actions between therapy sessions [10-12]. Research on caregivers’ adherence to therapy practice includes various methods of tracking practice, such as caregivers’ diaries, paper logs, and recall.

To effectively address factors that influence caregiver practice, therapists must develop a reliable method for recording practice that does not impose an excessive burden on caregivers. While previous studies on engagement have primarily focused on caregiver behavior during therapy sessions, there is limited research examining caregiver actions between sessions and how these relate to child characteristics, caregiver factors, and service delivery contexts [1,2]. Doing so will equip therapists to provide individually targeted care and promote improved child and family progress [1,2]. Researchers must consider assessing the methods used in other fields where tracking behavioral change outside of direct interaction is important, such as psychology and health and wellness coaching. One method frequently used in studies to promote changes in daily behavior is ecological momentary assessment (EMA). EMA is a process by which users respond to surveys or prompts about their behaviors in the moments those behaviors occur, using smartphones or other technological methods [13]. While EMA has not previously been used in early intervention (EI) programs, it has been applied in adjacent fields including physical activity, pain and surgical recovery, community participation, and parenting behaviors [14-19].

As an exploratory pilot study, this investigation aims to establish proof-of-concept for EMA methodology in pediatric therapy settings. The purpose of this pilot study is threefold: (1) to assess the feasibility and acceptability of EMA methodology for measuring caregiver practice in pediatric therapy, (2) to quantify the discrepancy between caregiver-recalled and EMA-documented practice, and (3) to identify factors that caregivers report as enhancing their practice opportunities.

## Methods

### Study Design

The initial phase of this pilot prospective cohort study involved developing and distributing a caregiver survey to collect demographic information about each child and their family, as well as caregiver factors that might influence practice. We selected potential study measures based on influences on caregiver engagement reported in previous studies [2,8,20]. Following the initial confidential Qualtrics survey, which was completed during September 2024, we offered all 34 participants the opportunity to contact the research team to volunteer for further participation in a 30-day EMA study. Five participants responded favorably and were trained on how to download the program and respond to prompts. During the prospective part of the study, participants received 1 daily prompt delivered via push notification to their smart devices at a personalized time (selected during enrollment to coincide with typical evening routines, most commonly between 7 PM and 9 PM). Single daily prompts were chosen to minimize participant burden while capturing retrospective daily practice, balancing feasibility with data completeness based on EMA design principles. Participants had the option to complete the survey immediately or “snooze” it to a later time of their choosing. This daily EMA prompt allowed respondents to report information about bouts and minutes of practice, as well as facilitators and barriers to that practice. Practice days refer to days on which caregivers responded “Yes” to practicing therapeutic activities and completed follow-up questions regarding practice frequency and duration. “All days” refers to the entire 30-day observation period, including both practice and nonpractice days, with nonresponse days coded as zero practice based on caregiver interview data. In this way, a balance between data capture and participant burden was achieved. After completing 30 days of EMA, during October 2024 and November 2024, caregivers had the option to provide feedback on the EMA process and their practice between therapy sessions in a single interview. Feasibility outcomes included EMA completion rate, retention, caregiver-reported burden, and follow-up interview feedback regarding usability and barriers to reporting. Because this was an exploratory pilot study, formal feasibility thresholds were not prespecified.

### Ethical Considerations

This study was approved by the Wichita State University Institutional Review Board (approval: 5752). Written informed consent was obtained from all participants prior to enrollment. Survey and EMA data were deidentified before analysis and stored on password-protected systems accessible only to the research team. Participants did not receive financial compensation for their participation.

### Participants

Recruitment occurred through pediatric therapy centers, public school programs, and EI programs in Kansas, as well as through social media recruitment. Participants were caregivers of children receiving occupational therapy, physical therapy, and speech-language pathology services across community-based and EI settings. Exclusion criteria included caregivers who did not have access to smartphones with iOS or Android operating systems, were unable to read English at a 6th-grade level or higher (as required for survey comprehension), or lacked basic smartphone app navigation skills (assessed through demonstration during enrollment). A total of 34 caregivers completed the initial survey. Of these, 5 volunteered to continue through the EMA process and completed a total of 30 days of EMA logging (). The final sample thus consisted of 5 participants, with data collected across 30 days each, resulting in a total of 150 data points.

### Variables

The primary outcome was the total minutes of caregiver-reported practice per day, calculated as the product of bouts per day and minutes per bout. Although adherence to prescribed therapy dosage is often used as an outcome in rehabilitation research, adherence could not be directly measured in this study because no standardized or consistently prescribed home program dosage was provided across participants. Instead, total daily practice time was selected as a pragmatic proxy for caregiver-delivered dosage exposure, reflecting the amount of therapeutic activity the child experienced between formal therapy sessions.

In the initial survey, caregivers were asked to recall the number of practice bouts they completed in the previous week, as well as the length of time for each practice bout. This value is referred to as “recall” practice dosage because it reflects the amount of practice caregivers reported doing without daily logging. Actual daily bout frequency and duration (in min) were recorded using ExpiWell (EMA) software as “prospective reporting.” The outcome variable was minutes of therapy per day, a product of bouts per day and minutes per bout. When participants did not complete their practice logs, zero minutes of practice were recorded for that day, a finding we confirmed during the follow-up caregiver interviews. Recall data reflected caregiver estimates from the week preceding EMA enrollment, whereas EMA data were collected prospectively during the subsequent 30-day observation period. During follow-up interviews, caregivers consistently reported that days without EMA entries generally corresponded to days when therapeutic practice did not occur; therefore, missing entries were coded as zero practice for descriptive analyses.

### Study Measures

Caregivers’ work schedules (full-time or part-time), the number of additional children in the home younger than the age of 18 years, the age of the child receiving the intervention, and the frequency of therapy sessions (2 vs ≥4 visits per month) were included as explanatory variables. Additional information collected included whether the caregiver remembered the therapist’s coaching, whether the session was fun for the child and caregiver, and the caregiver’s motivation level. Practice frequency (bouts per day) and practice duration (minutes per bout) were examined as secondary descriptive outcomes to provide additional context regarding caregiver practice patterns.

### Qualitative Methods

Follow-up interviews were conducted using a semistructured interview guide (Multimedia Appendix 1) immediately following the completion of the EMA period. Interviews were audio-recorded and transcribed for analysis. The primary investigator and physical therapy students, who received training in qualitative research methods, independently reviewed transcripts and interview notes using a descriptive content analysis approach. Initial codes were developed from recurring concepts related to caregiver practice, barriers, facilitators, and EMA usability. Codes were subsequently reviewed, refined, and organized into broader thematic categories through discussion and consensus with the primary investigator. Because the qualitative component served a supportive role within this exploratory pilot study and included only 5 participants, formal qualitative saturation was not an objective of the analysis. Rather, the qualitative findings were used to provide contextual information, enhance understanding of caregiver experiences, and assist in the interpretation of the quantitative EMA results.

### Data Analysis

Summary statistics, including means and SDs, were calculated for the outcome variables using the commercial software SAS 9.4. The low number of participants (n=5) completing the daily EMA logging limited the appropriateness of regression-based modeling; therefore, descriptive statistics are included instead, consistent with the pilot nature of this study.

Because missing EMA entries were coded as zero practice based on caregiver reports during follow-up interviews, a descriptive sensitivity analysis was conducted comparing EMA-reported practice across all study days with EMA-reported practice restricted to only the completed reporting days.

## Results

### Feasibility and Acceptability of EMA

**Figure 1.**
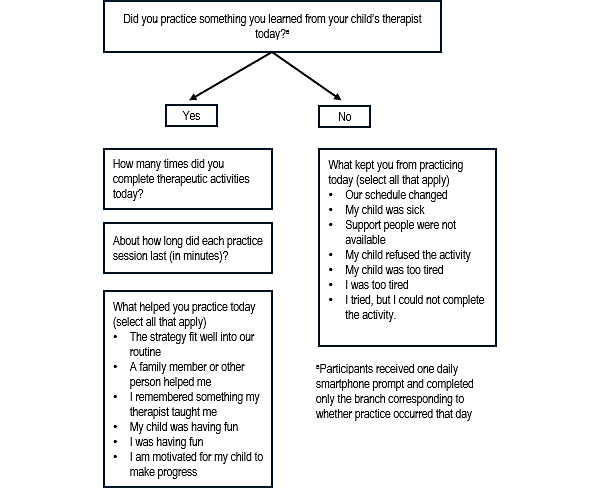
Ecological momentary assessment (EMA) survey structure and branching logic. Participants received 1 daily smartphone prompt. Depending on whether practice occurred, caregivers completed questions regarding practice frequency, duration, facilitators, or barriers to practice.

**Figure 2.**
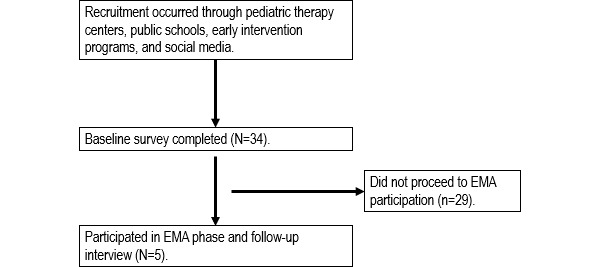
Participant flow diagram. EMA: ecological momentary assessment.

Participants completed 55% (82/150) of daily surveys (Figure 1). All participants took part in follow-up conversations regarding their experiences with the EMA process (Figure 2). Caregivers generally described EMA prompts as brief and manageable, although major life events and competing caregiving demands contributed to reduced adherence among some participants.

### Participant Characteristics and Practice Patterns

Compared with noncompleters, EMA completers demonstrated slightly higher baseline therapy frequency and reported daily practice, whereas educational attainment was similar between groups (Table 1). These comparisons were interpreted descriptively due to the small number of EMA completers. Demographics for the 5 caregivers are presented in Table 2. Child diagnoses varied in type and severity, and all caregivers had more than one child in the household. Participants included caregivers of children receiving occupational therapy, physical therapy, and speech-language pathology services. A total of 60% (3/5) of caregivers worked full-time, while the remaining 40% (2/5) worked part-time. Therapy frequency ranged from 1 to 3 sessions per week.

**Table 1.** Baseline characteristics of ecological momentary assessment (EMA) completers and noncompleters (N=34)^a^.

Characteristic	EMA^b^ completers (n=5)	EMA noncompleters (n=29)
Children in the home, mean (SD)	2.8 (2.1)	2.0 (1.0)
Therapy sessions/week, mean (SD)	1.8 (0.8)	1.5 (0.9)
Recalled practice bouts/day, mean (SD)	3.9 (5.8)	3.3 (3.8)
Recalled bout length (min), mean (SD)	15.6 (10.2)	20.2 (19.2)
Recalled total practice (min/d), mean (SD)	71.1 (120.9)	59.7 (73.9)
Full-time employment, n (%)	3 (60)	11 (37.9)
Bachelor’s degree or higher, n (%)	1 (20)	6 (20.7)

a Comparisons are descriptive only due to the small number of EMA completers.

b EMA: ecological momentary assessment

**Table 2.** Individual participant characteristics and comparison of caregiver recall and ecological momentary assessment (EMA)–reported practice by participant.

ID	Work	Children in home	Diagnosis of child of interest	Age of child of interest (y)	Frequency of therapy	Days logged (% of 30 total days)	Measurement comparison	
							Recall	EMA-reported
1	Full time	2	Delays in feeding and language	3	2x/week (8 times per month)	26% (8/30)	0.43 bouts/day 15 min/bout	0.20 bouts/day 7.2 min/bout
2	Full time	5	Cerebral palsy, GMFCS^a^ Level V	2	3x/week (12 times per month)	63% (19/30)	14.3 bouts/day 20 min/bout	2.57 bouts/day 7.6 min/bout
3	Full time	5	Global developmental delay	5	1x/week (4 times per month)	67% (20/30)	2.14 bouts/day 3 min/bout	5.57 bouts/day 3.7 min/bout
4	Part time	2	Cerebral palsy, GMFCS level III	6	3x/week (12 times per month)	70% (21/30)	4.29 bouts/day 10 min/bout	4.30 bouts/day 5.8 min/bout
5	Part time	3 (infant sibling born during study period)	Cerebral palsy, GMFCS level II	5	1x/week (virtual) (4 times per month)	47% (14/30)	1.43 bouts/day 10 min/bout	0.63 bouts/day 7.3 min/bout

a GMFCS: Gross Motor Function Classification Scale

Sensitivity analyses comparing completed EMA days with all study days demonstrated similar overall patterns of short, frequent practice bouts. On days when practice occurred, caregivers reported facilitators of practice using the EMA survey (Figure 1). The most frequently reported facilitators included recalling therapist coaching (40/82, 48.8% days), strategies fitting into daily routines (37/82, 45.1%), and the caregiver’s motivation to support the child’s progress (29/82, 35.4%). Less frequently reported factors included activities being fun for the child (24/82, 29.3%), fun for the caregiver (11/82, 13.4%), and support from another family member (5/82, 6.1%). Two participants experienced significant family events during the study, resulting in an abrupt decline in reporting after an initial period of consistent logging (Table 2).

### Practice Frequency and Duration

Group-level practice data are summarized in Table 3. Caregiver recall indicated higher average daily practice (4.5 bouts/d; 11.6 min/bout; 71.2 min/d) compared to EMA-reported practice averaged across all days (2.7 bouts/d; 6.5 min/bout; 23.2 min/d). Variability in recalled practice was high, particularly for total daily minutes (SD=121.0).

EMA-reported practice restricted to days when practice occurred demonstrated a higher frequency of bouts (5.2 bouts/d), but shorter duration (6.5 min/bout), resulting in lower total daily practice (23.9 min/d) compared to recall estimates.

**Table 3.** Comparison of caregiver recall and ecological momentary assessment (EMA)–reported practice, including sensitivity analysis for missing EMA data. EMA practice (practice days only) reflects practice recorded on days when it occurred. EMA practice (all days) includes all recorded days, with nonresponse days coded as zero practice based on caregiver reports. High variability in recalled practice suggests inconsistency in retrospective estimates.

Variable	Recalled practice (n=5), mean (SD)	EMA practice (practice days only; n=82 days), mean (SD)	EMA practice (all days; n=150), mean (SD)	Mean difference (recall–EMA all days)^a^
Daily practice (number of bouts)	4.52 (5.65)	5.20 (3.28)	2.65 (4.39)	1.87
Bout length (min)	11.60 (6.35)	6.48 (6.45)	6.48 (6.45)	5.12
Total daily practice (min)	71.19 (121.02)	23.93 (14.72)	23.19 (14.12)	48.00

a Mean difference scores are presented descriptively because of the exploratory pilot nature of the study.

### Comparison of Recall and EMA by Participant

**Figure 3.**
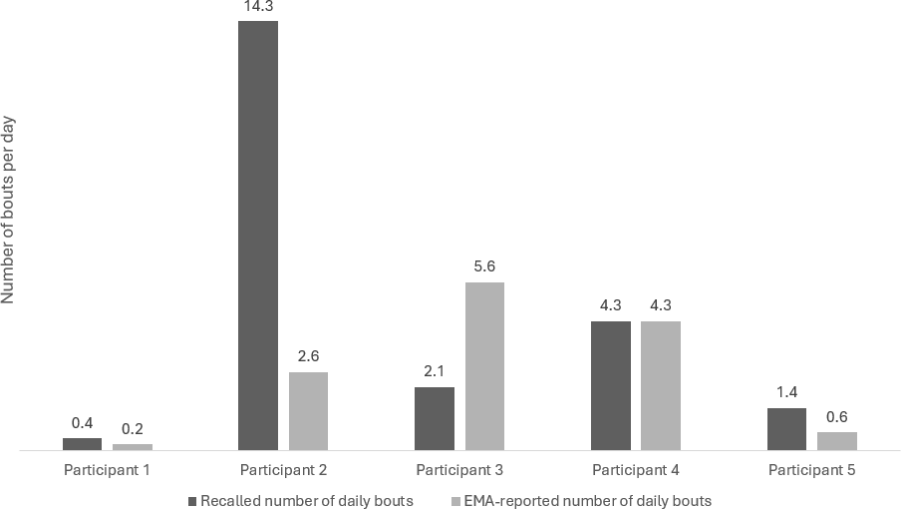
Comparison of caregiver recall versus ecological momentary assessment (EMA)–reported number of daily practice bouts by participants (individual data). EMA values reflect averages across all recorded days. Substantial variability is observed between recall and prospectively recorded practice.

Caregiver recall and EMA-reported practice differed across participants in both the frequency and duration of practice bouts (Table 2; Figures3  4). Caregivers generally reported a higher number of daily bouts and longer bout durations than those captured via EMA, although discrepancies varied in magnitude and direction.

For example, Participant 2 demonstrated a substantial overestimation in both frequency (14.3 vs 2.57 bouts/day) and duration (20.0 vs 7.6 min/bout), whereas Participant 3 reported fewer bouts and shorter durations via recall compared to EMA (2.14 vs 5.57 bouts/day; 3.0 vs 3.7 min/bout).

When combined, these differences resulted in consistently higher recalled estimates of total daily practice relative to EMA. Overall, discrepancies between recall and EMA were variable across participants but tended to overestimate total practice. These patterns suggest that discrepancies between recall and EMA arise from differences in both the reported frequency and the duration of practice.

**Figure 4.**
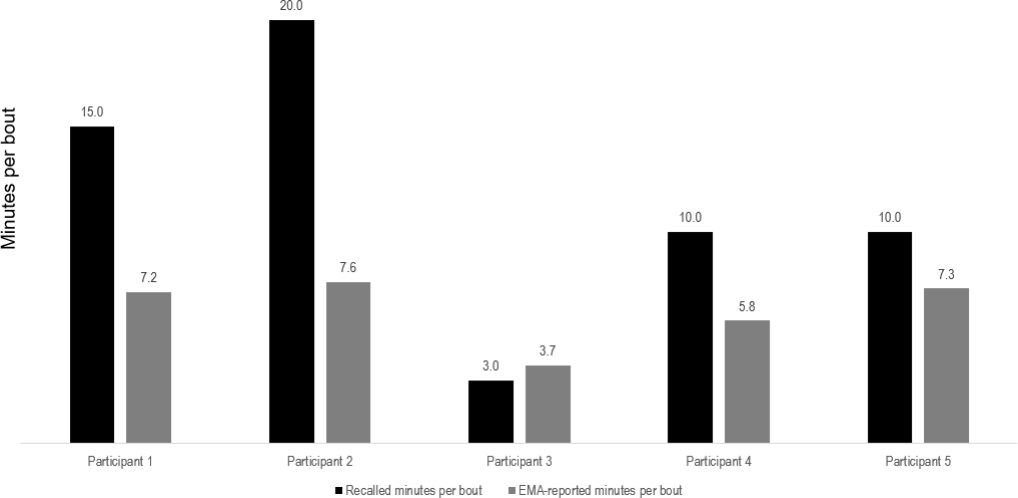
Comparison of caregiver recall versus ecological momentary assessment (EMA)–reported minutes per bout by participant (individual data)*.*

## Discussion

### Caregiver Practice Patterns

The aim of this pilot study was to use EMA to measure the daily practices of caregivers of children with disabilities and to compare recalled practices with prospectively reported practices over a 30-day period. Caregivers in our study practiced multiple times per day for short periods of time. On days when practice occurred, participants engaged in approximately 4 to 6 bouts per day, each lasting 5 to 7 minutes (Table 2), suggesting that practice is distributed throughout the day and integrated into daily routines rather than occurring in extended sessions.

This pilot study suggests that EMA may provide useful information regarding caregiver-reported therapy practice in pediatric rehabilitation settings. Although caregivers generally described the prompts as manageable, substantial attrition and moderate completion rates indicate significant feasibility challenges. Follow-up interviews highlighted the influence of competing family demands and major life events on EMA adherence, emphasizing the challenges of longitudinal caregiver reporting in real-world contexts Table 4.

**Table 4.** Caregiver-reported feasibility and practice themes identified during follow-up interviews.

Theme	Representative caregiver observations
Practice embedded within routines	“Caregivers described how embedding the therapeutic activities into functional activities they did on a daily basis (dressing, mealtimes, toileting activities, getting onto and off of surfaces like wheelchairs, chairs, tub benches, and standing) made the therapeutic activities much easier to practice and for longer periods of time.”
Impact of making the therapeutic activities fun for the child	“Caregivers described how making the therapeutic activities fun and engaging for the child encouraged daily practice, longer practice, and recording practice.”
Impact of family life events on EMA^a^ completion	“Caregivers described surgery, birth of a sibling, and competing caregiving responsibilities as barriers to EMA completion and daily practice reporting.”
Impact of the number of therapy visits	“Across all caregivers, the higher the level of involvement in formal therapeutic sessions with the therapists increased the number and length of caregiver action between formal sessions.”
EMA usability and burden	“Caregivers generally described EMA prompts as brief and manageable within daily routines, although several reported forgetting to complete entries on days when practice did not occur.”
Caregiver motivation and concern	“Caregiver concern about their child’s progress and recovery appeared to influence therapeutic activity frequency and consistency.”
Child and family involvement	“Several caregivers described siblings and other family members assisting with practice activities, particularly in children with more complex needs. Involving the whole family into therapeutic activity practice added to both the fun and success!”
Lack of impact of making the therapeutic activities fun for the caregiver	“Caregivers noticeably did not describe or record their own enjoyment as important to practice between therapy sessions.”

a EMA: ecological momentary assessment.

Caregiver recall differed substantially from EMA-reported practice. Recalled estimates were highly variable and generally higher than prospectively reported values, particularly for total daily practice. Discrepancies varied across participants in both magnitude and direction, indicating inconsistent recall of both frequency and duration. Together, these findings suggest that retrospective caregiver reports may differ from prospectively documented daily practice.

An alternative explanation for some of the observed discrepancies is that caregiver practice may have genuinely changed between the recall period and the subsequent EMA observation period. Follow-up interviews revealed that several caregivers experienced major life events, including surgery, the birth of a sibling, and competing caregiving responsibilities, which influenced both practice opportunities and reporting behavior. These qualitative findings suggest that at least part of the difference between recall and EMA estimates may reflect real fluctuations in caregiver practice over time rather than measurement differences alone. Consequently, the present findings should be interpreted as reflecting both potential recall inaccuracy and the dynamic nature of caregiver-delivered practice within changing family contexts.

### Comparison With Other Literature

The EMA completion rate in this study was 55%. Although this completion rate is lower than the 70% to 80% response rates often reported in controlled EMA studies, adherence to EMA protocols varies substantially depending on study duration, prompt frequency, participant burden, and participant characteristics [14,16-19]. Participants in this study were caregivers of children with disabilities who frequently described competing family demands and major life events as barriers to reporting. The decline in reporting observed following significant family events is consistent with prior EMA literature demonstrating challenges with sustained engagement in real-world settings [14,16-19]. While participants generally described the EMA prompts as brief and manageable, the observed completion rate and substantial attrition between survey completion and EMA participation suggest that EMA may be feasible for some caregivers but may also present participation barriers for others.

These findings are consistent with the literature on caregiver engagement in pediatric rehabilitation, which suggests that the implementation of home-based strategies is shaped by multiple interacting factors, including motivation, perceived relevance, and integration into daily routines [7,8,20]. Caregiver-reported facilitators in this study—such as routine-based strategies, child engagement, and recall of therapist guidance—reflect these influences and align with existing work emphasizing the importance of caregiver knowledge, attitudes, and context in shaping adherence [7,21-24].

### Routines-Based Interventions

Consistent with existing literature, integrating intervention strategies into daily routines appears to support caregiver implementation of practice [6,10,11,25]. Caregivers described embedding strategies into activities such as meals, play, and community participation. These findings align with prior research demonstrating that routines-based interventions may support adherence and functional outcomes [7,26,27]. Although much of this work has focused on EI settings, our findings suggest that routines remain relevant across service delivery contexts.

### Other Facilitators of Practice

Caregiver interviews highlighted the importance of a child’s enjoyment in supporting practice. Strategies perceived as fun for the child were associated with increased engagement, consistent with literature emphasizing interest-based interventions and the role of enjoyment in promoting participation [25,28,29]. Caregivers also frequently reported applying therapist guidance during daily routines. Although not defined in detail within the EMA survey, this finding highlights the potential importance of effective caregiver coaching strategies, including modeling, practice, and feedback, in supporting carryover between sessions [7,10].

### Role of Therapy Frequency

Caregivers in this study received relatively frequent therapy services, with most participants attending 2‐3 sessions per week. These caregivers also described high levels of engagement in practice and, in some cases, active advocacy for increased services. The co-occurrence of frequent services and caregiver-reported practice suggests that therapy dosage and caregiver implementation may be interrelated. These preliminary findings suggest that therapy frequency, routine integration, and child engagement may influence caregiver practice, although these relationships require confirmation in larger studies.

### Limitations

This pilot study has several important limitations. The small sample size and high attrition rate limit generalizability and highlight feasibility challenges for longitudinal EMA studies in caregiver populations. Selection bias is likely, as only 5 of 34 caregivers who completed the initial survey elected to participate in the EMA phase. Caregivers willing to engage in 30 days of smartphone-based reporting may differ systematically from those who declined participation, potentially representing families with greater motivation, interest in therapy, comfort with technology, or capacity to accommodate additional study demands. Consequently, the findings may not reflect the experiences of all caregivers of children receiving therapy services. Additionally, the small sample exhibited substantial heterogeneity in child diagnoses, family structure, therapy frequency, and life circumstances (for example, surgery or the birth of a sibling), which may have influenced caregiver practice patterns and limited interpretation of group-level averages. Because recall and EMA reporting occurred during different time periods, differences between methods may partially reflect temporal variation in caregiver practice rather than differences attributable solely to the reporting method.

Although educational attainment appeared similar between completers and noncompleters, the substantial attrition between baseline survey completion and EMA enrollment suggests that unmeasured participation barriers may have influenced study involvement. These barriers may include competing caregiving responsibilities, technology demands, time constraints, caregiver burden, or other factors not captured in the baseline survey. Consequently, the findings may primarily reflect the experiences of caregivers who were willing and able to participate in daily EMA reporting.

Participants who completed the EMA phase demonstrated somewhat higher baseline therapy frequency and recalled practice than noncompleters, suggesting possible selection bias toward caregivers who were already highly engaged in therapeutic activities. Because missing EMA entries were coded as zero practice based on caregiver reports during follow-up interviews, a descriptive sensitivity analysis was conducted comparing EMA-reported practice across all study days with EMA-reported practice restricted to completed reporting days only. Since EMA prompts were delivered once daily in the evening, responses reflected same-day retrospective reporting rather than true real-time momentary assessment.

The 55% EMA completion rate introduces potential missing data bias, and reliance on self-report limits the ability to verify the quantity or quality of caregiver-delivered practice. Although missing EMA entries were coded as zero practice based on caregiver reports during follow-up interviews, it is possible that some instances of practice were not recorded, which may have led to an underestimation of caregiver practice. Caregiver psychological factors, which may influence practice behavior, were not systematically measured and represent a potential source of unmeasured confounding.

Child outcomes were not assessed, precluding the examination of the relationship between caregiver practice dosage and developmental progress. Finally, the 30-day observation period may not capture longer-term patterns of caregiver behavior or changes in engagement over time.

### Areas for Future Research

Future research should include larger and more diverse samples to validate these findings and examine relationships between caregiver practices and child outcomes. Incorporating measures of caregiver psychological state, including fatigue, stress, and mood, may further clarify factors influencing practice behavior, particularly given the physical and cognitive demands of caregiver-delivered interventions [30]. Efforts to improve EMA feasibility, such as reducing burden or enhancing engagement, will also be important. Additional work is needed to better understand how caregiver characteristics, child factors, and service delivery interact to shape practices between therapy sessions.

### Conclusions

This pilot study demonstrates that caregiver-delivered practice occurs in short, frequent bouts embedded within daily routines. Caregiver recall differed significantly from prospectively reported EMA data, suggesting that retrospective recall and prospectively reported EMA data may differ substantially. Findings highlight the potential importance of embedding intervention strategies into routines and promoting child engagement to support caregiver implementation. EMA provided useful information regarding caregiver practice among participating caregivers, although substantial attrition and moderate completion rates indicate significant feasibility challenges that should be addressed in future studies.

## Supplementary material

10.2196/83548 Multimedia Appendix 1 Initial Qualtrics survey and follow-up interview questions.

## Abbreviations

EI: early intervention
EMA: ecological momentary assessment
