# Supplementary material for: Using Ecological Momentary Assessment to Document and Investigate Caregiver Practices Between Pediatric Therapy Sessions: Prospective Pilot Cohort Study
Source: JMIR Form Res. 2026 Jul 3;10:e83548. doi: 10.2196/83548 (PMC13331399; doi:10.2196/83548)
Supplement: Multimedia Appendix 1 [file formative-v10-e83548-s001.docx]

**Multimedia Appendix 1**

**Addendum #1. Initial Qualtrics Survey**

We are a group of researchers from the Wichita State University Department of Physical Therapy and the Family, Infant, and Preschool Program (FIPP). We are contacting you because you are the caregiver of a child receiving FIPP services. We are recruiting research participants to help us explore how caregivers use strategies they learn in early intervention between sessions. Participation involves completing a survey that will take about 15 minutes.   In addition to the survey questions, we will request some basic family demographic information.

There are no personal benefits or anticipated risks to participating in this study. However, if you feel uncomfortable with a question, you may skip it. Participation is voluntary, and you can stop taking the survey at any time.

 We will work to make sure that no one sees your survey responses without approval. But, because we are using the Internet, there is a chance that someone could access your online responses without permission. In some cases, this information could be used to identify you.

 If you have any questions, please contact Jennifer James at Jennifer.James@wichita.edu.

For questions about the rights of research participants, you may contact the Office of Research and Technology Transfer at Wichita State University, 1845 Fairmount Street, Wichita, KS 67260-0007, and telephone (316) 978-3285.

 You are under no obligation to participate in this study.  By selecting “Yes” below, you are indicating that:

 •     You have read (or someone has read to you) the information provided above,

•     You are aware that this is a research study,

•     You have voluntarily decided to participate.

_____ Yes ______No (If no, survey ends with a “thank you for your time” response.)

|  |  |
| --- | --- |

I am age 18 or over.
_____ Yes ______No (If no, survey ends with a “thank you for your time” response.)

I would like to have a copy of the Consent Form emailed to me.

_____ Yes ______No

*Consider the child who is receiving physical, occupational, or speech therapy.*

Q1. What is the work schedule like for the *primary* caregiver of this child (select one)?

_____ Full time caregiver

_____ Work from home (full or part-time)

_____ Work outside the home (part-time (up to 30 hours))

_____ Work outside the home (full-time (31 hours or more))

Q2. What is the schedule of the *secondary*caregiver for this child (select one)?

_____ Full time caregiver

_____ Works from home (full or part-time)

_____ Works full or part-time outside the home

_____ Child has no secondary caregiver

Q3. How many children live in your home at least half the time (please write a whole number, below)?

____________________

Q4. What is the primary caregiver's highest level of education?

_____ Some high school

_____ High school grad/GED

_____ Some college/technical school

_____ College degree or higher

Q5. How many scheduled visits does your child have with FIPP per month (please enter a whole number)?

| ____________________ |  |
| --- | --- |

Q6. On average, how many minutes is each therapy session your child attends?

Participant was given a sliding scale ranging from 0-100 “minutes per session.”

Q7. In the past week, about how many times did you use strategies you learned from your FIPP provider with your child?

Participant was given a sliding scale ranging from 0-100 *“Number of practice times.”*

Q8. When you practice a strategy with your child ***outside*** of PT, OT, or speech, about how many minutes do you spend?

Participant was given a sliding scale ranging from 0-100 *“Minutes spent per practice.”*

We are doing a follow-up for families to log the strategies they use at home on an app to help them work practice into their days. These logs will take no more than 2 minutes per day and will help us understand when and how caregivers use strategies they learn from their child's therapist. If you would be interested in hearing more about this please talk with your FIPP provider. You may also share your email here, and we will prompt your FIPP provider to follow-up with you about the second phase of this project.

**Addendum 2. Follow up interview questions**

1. How did the amount of practice you did, match what you expected?
2. Tell me about things that made you more likely to practice…(the following prompts can be used)
   1. What are some routines that made practice easier?
   2. What are some strategies that made practice easier?
   3. What are some external supports that made practice easier?
3. Tell me about things that made you less likely to practice…(the following prompts can be used)
   1. What are some routines that made practice harder?
   2. What are some strategies that made practice harder?
   3. What are some external interference that made practice harder?
4. Tell me how important you think practice is to your child’s progress…(the following prompts can be used)
   1. What changes do you see when you practice more vs. when you practice less
   2. What do you think drives your child’s progress the most?
5. Tell me how the process of daily surveys impacted your practice…
